# Coexistence of Lichen Planus Pigmentosus and Classic Lichen Planopilaris: A Case Report and Literature Review

**DOI:** 10.7759/cureus.46952

**Published:** 2023-10-13

**Authors:** Rana A Almarek, Nada G AlQadri, Manar Alotaibi

**Affiliations:** 1 Department of Dermatology, King Saud Medical City, Riyadh, SAU; 2 Department of Dermatology, Prince Sultan Military Medical City, Riyadh, SAU

**Keywords:** frontal fibrosing alopecia, lichen planopilaris (lpp), lichen planopilaris, classic lichen planus, : lichen planus pigmentosus

## Abstract

Lichen planus (LP) is a common inflammatory skin disorder with multiple variants. The coexistence of lichen planus pigmentosus (LPPigm) and frontal fibrosing alopecia is well-established in the literature. However, the coexistence of LPPigm and classic lichen planopilaris (LPP) is rare. We report a case of LPPigm and classic LPP in a postmenopausal woman with a literature review.

## Introduction

Lichen planus (LP) is a chronic lichenoid inflammatory disorder that affects the skin, scalp, and/or mucous membranes. It has multiple clinical variants based on the morphology of the lesions and the site involved [1].

Lichen planus pigmentosus (LPPigm) is an uncommon variant of LP. It is characterized clinically by persistently acquired macular pigmentation ranging from dark brown to gray. It affects the sun-exposed regions of the face and neck as well as sun-protected areas of flexural skin, such as the axillae and inguinal regions in dark-skinned individuals [2].

Lichen planopilaris (LPP), a follicular form of LP, is a primary lymphocytic cicatricial alopecia that often manifests as vertex or parietal scalp hair loss. Lymphocytic infiltration leads to the destruction of hair follicles and, eventually, scarring of the scalp. Patients may also experience itching, burning, or tenderness of the scalp [3,4].

Lichen planus pigmentosus can present concomitantly with other variants of LP as frontal fibrosing alopecia (FFA) [2], and there have been a remarkably increasing number of cases of this association in the past decade. However, the coexistence of LPPigm and classic LPP is extremely rare.

We present a case of a 61-year-old female who developed both LPPigm and classic LPP.

## Case presentation

A 61-year-old postmenopausal woman presented to the dermatology clinic with a few weeks’ history of dark pigmentation over the sides of her neck. Lesions were asymptomatic, but she was primarily concerned about the cosmetic appearance. There was no history of new medications, excessive sun exposure, or topical application. Upon further questioning, she gave a history of hair loss over the crown area of the scalp that started as a single alopecic patch 20 years ago and progressed over time. She used to follow up inconsistently with different dermatologists for her scalp alopecia. However, she was unaware of the diagnosis and was started on topical medications, including corticosteroids, tacrolimus, and minoxidil solution. Among those medications, no oral therapy was given. Her family history and personal medical history were unremarkable.

**Figure 1:**
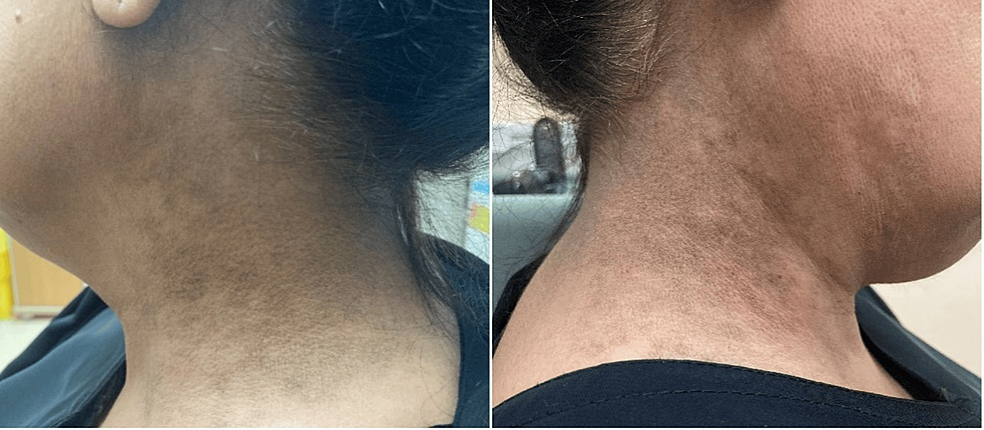
Bilateral ill-defined confluent gray-brown macules and patches in a reticular pattern over the sides of the neck.

The patient has a Fitzpatrick skin phototype IV. Her skin examination showed ill-defined gray-brown pigmented macules and patches in a reticular pattern distributed symmetrically over the sides of her neck (Figure 1).

**Figure 2:**
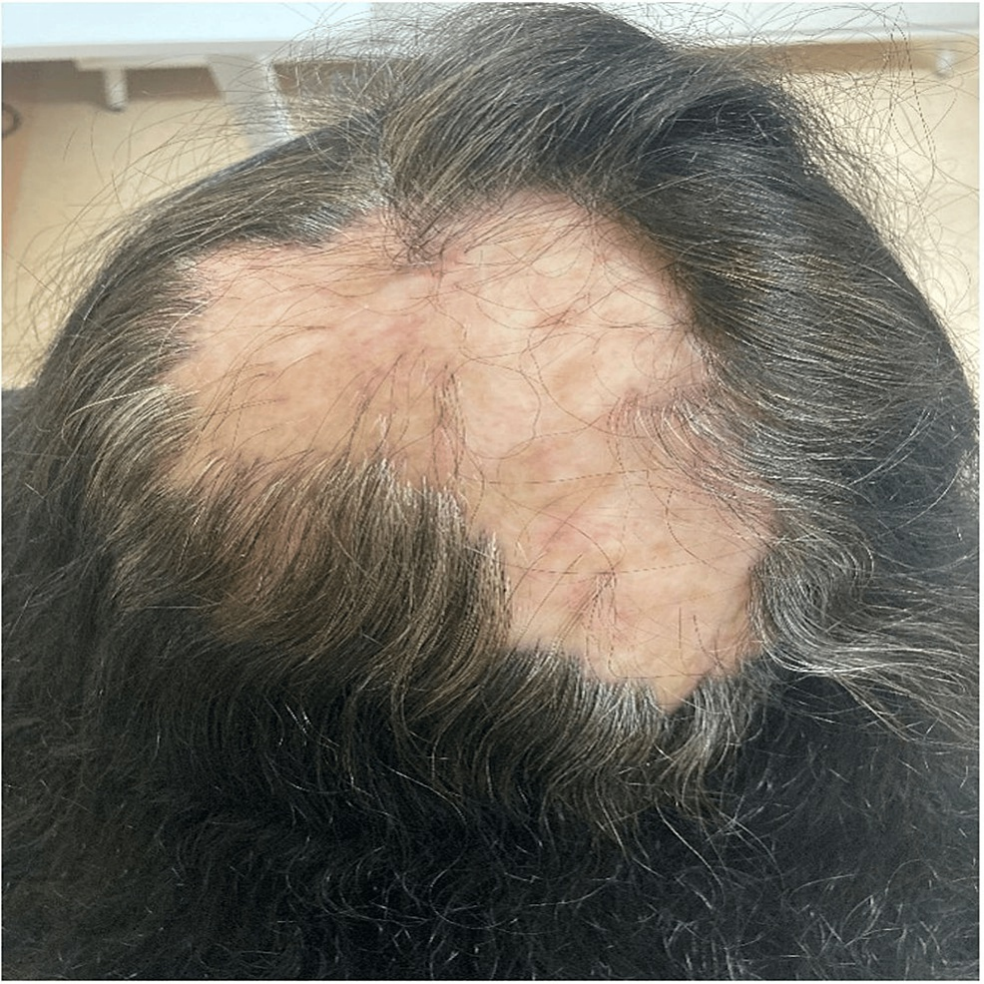
Single alopecic patch over the vertex of the scalp.

There was no involvement of the nails, oral, or genital mucosa. Other skin examinations were unremarkable except for tinea pedis over the fourth web space bilaterally. Hair examination showed a single scarring alopecic patch over the vertex area of the scalp measuring 14.5 x 8 cm (Figure 2).

**Figure 3:**
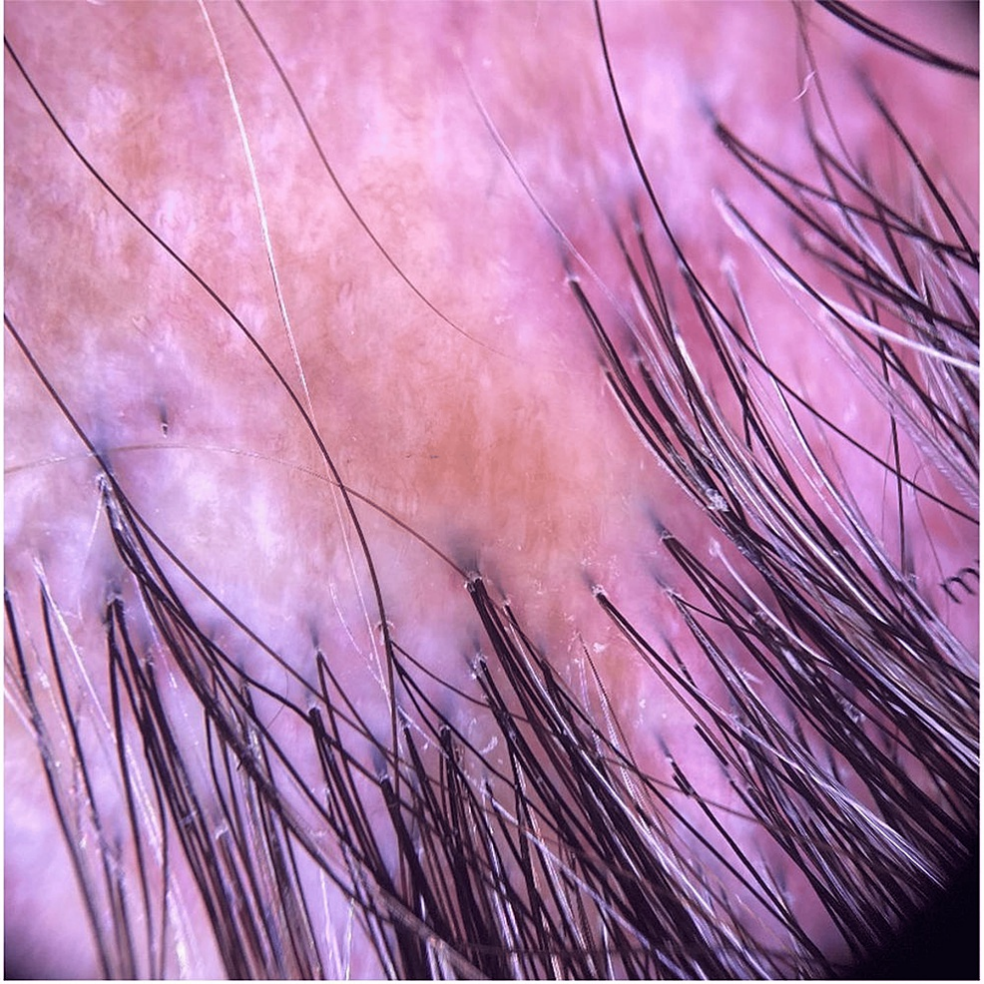
Dermoscopic features of the scalp show perifollicular scales, perifollicular erythema, scarred white areas, and loss of follicular ostia.

The hair pull test was negative. The frontotemporal hairline was preserved, and there was no loss of eyebrows or body hair. A dermoscopic examination of the margins of the expanding area of alopecia showed perifollicular scaling, perifollicular erythema, and loss of follicular ostia (Figure 3).

**Figure 4:**
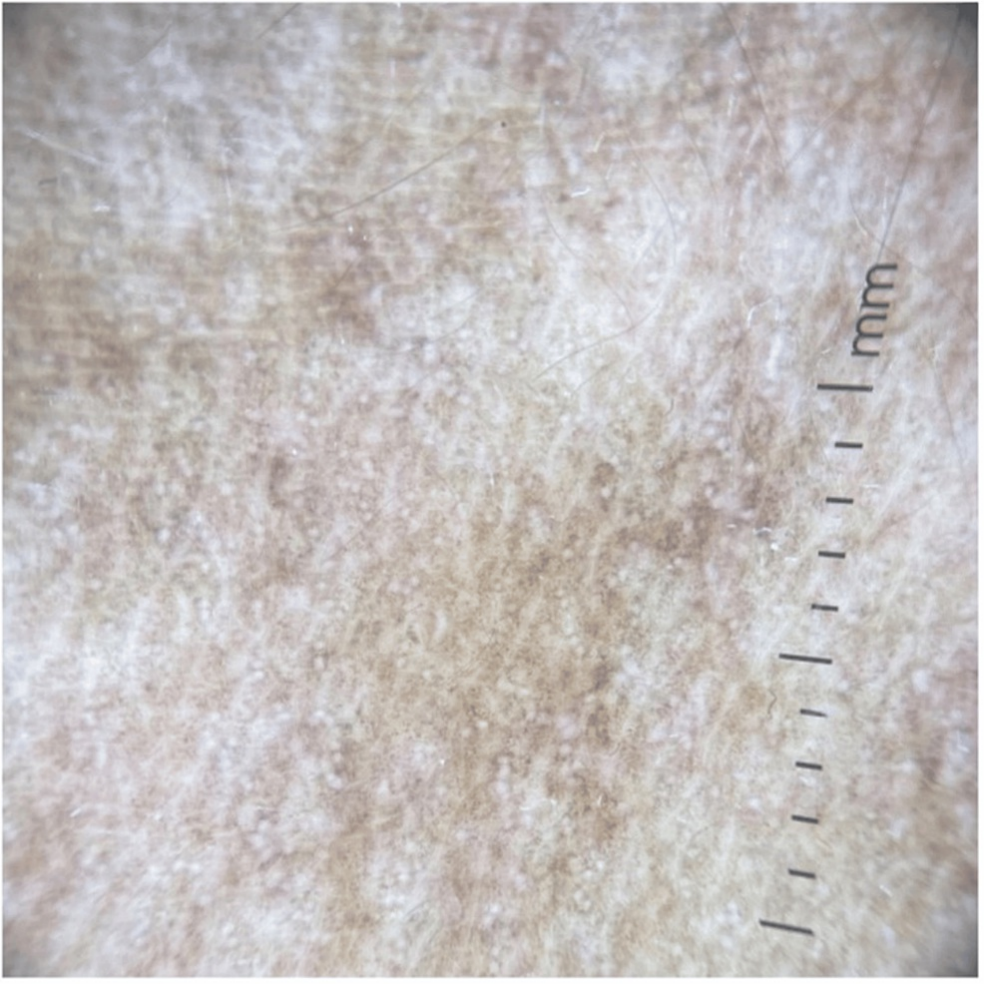
Dermoscopic features of neck pigmentation show speckled gray-brown dots and globules sparing necklines and follicular openings.

A dermoscopy of the neck hyperpigmentation showed speckled gray-brown dots and globules sparing necklines and follicular openings (Figure 4).

**Figure 5:**
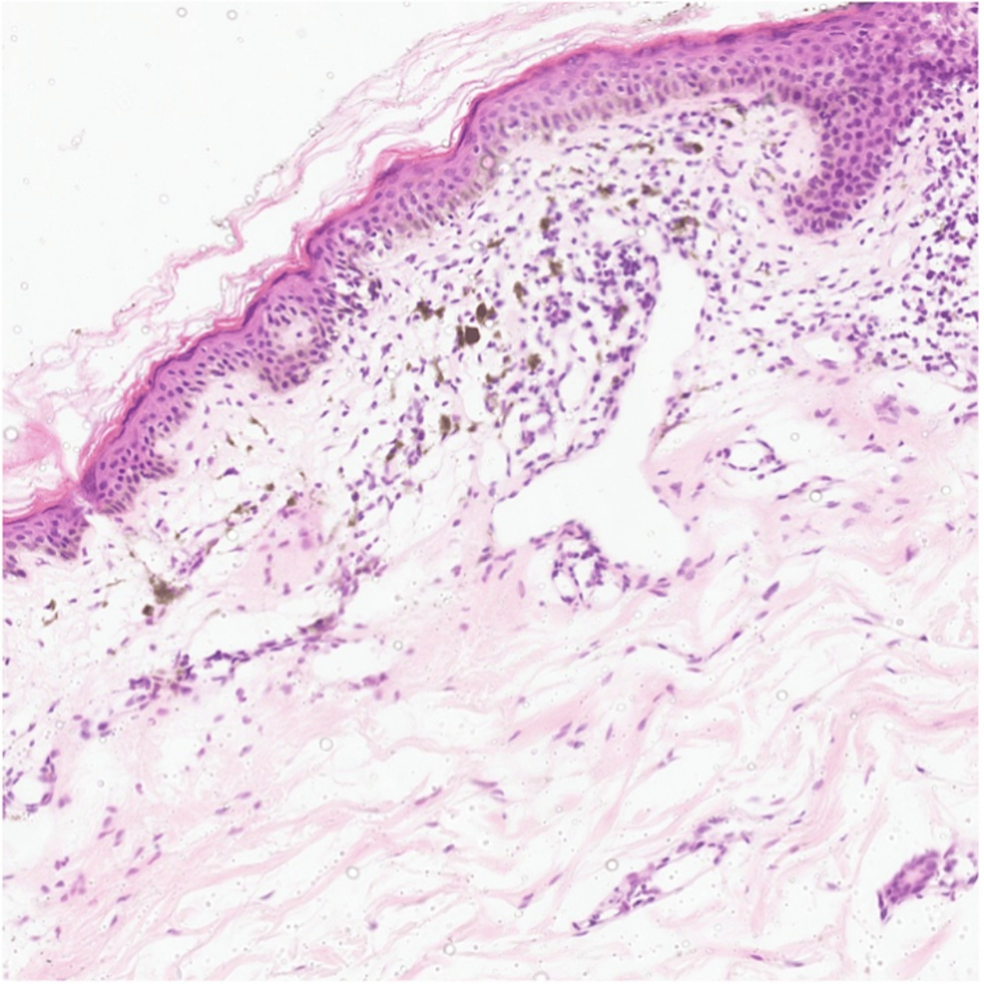
Histopathology of neck pigmentation shows epidermal atrophy, focal vacuolar degeneration, superficial dermal and perivascular lymphocytic infiltration, pigment incontinence, and melanophages.

Her laboratory results, including complete blood count with differential (CBC w/ diff.), urea and electrolytes (U/E), liver function test (LFT), hepatitis B and C serology, human immunodeficiency virus (HIV), and QuantiFERON-TB Gold test, were unremarkable. A 3-mm punch biopsy from the hyperpigmented lesions on the neck revealed focal vacuolar degeneration and lymphocytic dermal infiltrates, mainly perivascular, with pigment incontinence and melanophages, favoring the diagnosis of LPPigm (Figure 5).

**Figure 6:**
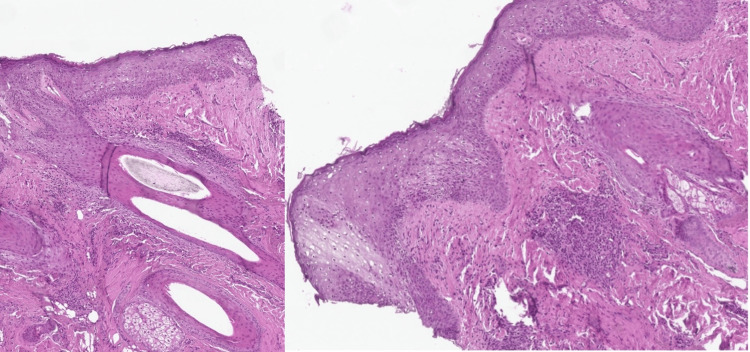
Histopathology of the scalp shows epidermal hypergranulosis, focal dermal and perifollicular lymphocytic infiltration, and dermal and perifollicular fibrosis.

Another 5-mm punch biopsy from the scalp showed mild epidermal hypergranulosis with rare follicular dyskeratosis, focal dermal lymphocytic infiltrate, and dermal and perifollicular fibrosis with no evidence of mucin deposition, favoring a diagnosis of LPP (Figure 6).

Based on the clinical, dermoscopic, and histological findings, the diagnosis of LPPigm and classic LPP was established. The patient was instructed about strict photoprotection and to use topical tacrolimus 0.01% ointment for the neck lesions along with topical minoxidil 5% solution, topical clobetasol propionate scalp lotion, and doxycycline 100 mg per oral (PO) twice a day (BD) for her scalp alopecia with a monthly intralesional injection of triamcinolone acetonide 10 mg/cc. We are still following up with the patient for a treatment response.

## Discussion

Lichen planus pigmentosus usually affects patients of skin types III to V, mainly type IV. [5] It is more commonly seen in females, with a peak age of onset of the disease between 30-39 years [6]. It is characterized by an insidious onset of ill-defined brown to gray macules that develop over sun-exposed areas, most commonly the face and the neck, which later progress into confluent hyperpigmented brown patches [5]. Lesions are asymptomatic but sometimes can be pruritic, with no history of previous inflammatory processes or erythema [5,6]. The most common pigmentation patterns are diffuse, reticular, blotchy, and perifollicular [6]. Our patient had a typical clinical presentation of LPPigm but a higher-than-average age at onset. Histopathologically, it is characterized by an epidermal basket weave horny layer with minimal change in epidermal thickness, keratinocyte apoptosis, and basal cell layer vacuolar degeneration. Dermal changes include the presence of band-like or perivascular lymphohistiocytic inflammatory infiltrates, scattered melanophages, and melanin incontinence. In older lesions, the epidermis is more atrophic, there is absent or focal vacuolar degeneration, and there is a prominence of perivascular lymphocytic infiltrates with more melanophages, as in our case [5]. Dermoscopic features of LPPigm may include a pseudo-network pattern, blue-grey dots arranged in circles, speckled blue-grey dots (which is the pattern seen in our patient), a dotted pattern, rhomboids with asymmetric pigmented follicular openings, and loss of facial vellus hair [7]. The dotted pattern was found to be a distinctive dermoscopic pattern found in patients with LPPigm associated with FFA [7,8]. 

Lichen planopilarisis is characterized by lymphocytic infiltration of hair follicles, leading to the development of cicatricial alopecia. It mainly affects middle-aged Caucasian females. Clinically, LPP is characterized by erythema of the scalp, perifollicular scaling, follicular hyperkeratosis, and a positive anagen pull test, which is the case in our patient. However, she had a negative pull test, which could be explained by decreased disease activity at the time of presentation. It is classified into three clinical variants: classic LPP, FFA, and Graham-Little-Piccardi-Lassueur syndrome. Classic LPP shows a single or multifocal area of irregular alopecia that is more common in the vertex or parietal region and without a characteristic band distribution. Our patient had the distribution of classic LPP. Frontal fibrosing alopecia occurs slowly in the frontotemporal hairline in a band-like distribution and is commonly associated with eyebrow alopecia [4,9]. In addition to the scalp, other manifestations may arise, such as LPPigm, facial papules, body hair involvement, hypochromic lesions, diffuse erythema on the facial and cervical regions, and evident frontal veins [9]. Lichen planopilarisis is characterized by perifollicular erythema, perifollicular desquamation, and loss of follicular orifices on trichoscopy [4]. In our patient, these trichoscopic findings were present at the active edge of the scalp lesion. Histopathologically, LPP is characterized by perifollicular lymphohistiocytic infiltrate, sometimes with a lichenoid pattern that is more prominent in the upper portion (isthmus and infundibulum regions), vacuolar degeneration of basal cells, necrotic keratinocytes, and artifactual clefts between the follicle and the perifollicular fibrous band; perifollicular fibrosis can be seen separating the inflammatory infiltrate from the follicle. Over time, there is a reduction and loss of sebaceous glands and the destruction of the entire hair follicle, which is replaced by connective tissue [9]. Our patient had mild epidermal hypergranulosis, focal dermal and perifollicular lymphocytic infiltrates, and mild dermal and perifollicular fibrosis, favoring the diagnosis of LPP.

The association between FFA and LPPigm is well-established in the literature. It was first described in 2013 by Dlova et al. in 24 patients in South Africa [10]. Since then, many articles have identified this association in English literature [7,11-18]. The largest study was a multi-centric retrospective study of 104 patients. In this study, the mean age was 60.5 years, and 97.1% of the patients were females. Most patients were postmenopausal, corresponding to the general FFA epidemiology [11]. The association of FFA with LPPigm was noticed to be more common in women of dark skin phototypes (IV-VI), in which LPPigm is more prevalent [7,10-12]. However, few articles reported this association in Caucasians or lighter skin phototypes (II and III). [13,15,16]. Most patients with this association were postmenopausal women [11,7,13]. However, in the case series by Dlova et al., an earlier age of onset was noticed (64% were premenopausal), which was attributed to the marked traction exerted by African women on their hairstyle, which ended up damaging the follicle early. The onset of hair loss and pigmentation was variable between studies; LPPigm can present before [10,11,13,14,16] or after FFA [12,15,17], or they can occur simultaneously [13, 18]. Lichen planus pigmentosus preceded the onset of FFA in many cases [10,11,13,14]. It has been suggested that LPPigm can be a warning sign of developing FFA, and clinicians should follow these patients closely for signs of FFA [10,11,14]. Furthermore, LPPigm may represent a poor prognostic factor for FFA [11]. Interestingly, face pigmentation is almost always affected in LPPigm patients with FFA. The recession of frontotemporal hairline and co-localization of pigmentation suggest a probable role of contact allergy due to hair dye or facial cosmetics in the pathogenesis of both conditions. This could be explained by the lesser density of hair in the anterior aspect of the scalp, as well as contact with a larger amount of hair dye product with the initial stroke of application, which results in preferential involvement of the anterior hairline through contact allergy as well as photosensitization [8]. Only one case had exclusive neck involvement without facial involvement [17], indicating a similar involvement in our patient. Few patients had characteristic periocular pigmentation of the upper eyelid. This could act as a clue to the diagnosis of facial LPPigm and help differentiate it from other dermatoses, such as melasma [16].

Although it was reported in the literature that classic LPP can develop in association with cutaneous LP [4], we found that the coexistence of classic LPP and LPPigm is extremely rare, with only a few cases reported in the literature [6,19,20]. Cobos et al. reported a premenopausal African American woman who presented with dark pigmentation on her arms, neck, and face that was diagnosed as LPPigm. The pigmentation was preceded by scarring hair loss that mainly affected the frontal scalp, with a few portions of her occipital scalp affected, which was diagnosed as LPP. Our patient had a similar presentation in which the LPP preceded the development of the pigmentation but with a much later onset and a lag time of 20 years. A clinical and epidemiological study of 124 Indian patients with LPPigm found only two patients with associated follicular LP, a synonym of LPP [6]. A case series of six patients with follicular LPPigm noticed the presence of LPP in the scalp of two patients, suggesting that follicular LPPigm is a macular form of LPP [20].

To the best of our knowledge, our patient is one of the few cases in the literature with this association.

## Conclusions

Lichen planus is a chronic inflammatory dermatological disorder with multiple clinical and morphological variants. The coexistence of its two clinical variants, LPPigm and FFA, is well-established in the literature. However, the coexistence of classic LPP and LPPigm is rare. This case report further illustrates the association between LPPigm and primary cicatricial alopecia in the LPP group, regardless of the clinical variant. Since both diseases are psychologically distressing, especially when coexisting, dermatologists should be aware of this association. Thus, for these patients who present with either LPPigm or LPP and its variants, it may be useful to evaluate them for either hair loss or pigmentation and follow them closely, as early recognition and treatment carry a better outcome.
